# Sod translocation to restore habitats of the myrmecophilous butterfly *Phengaris (Maculinea) teleius* on former agricultural fields

**DOI:** 10.1002/ece3.9293

**Published:** 2022-09-09

**Authors:** Cristina G. Sevilleja, Frank Van Langevelde, Juan Gallego‐Zamorano, Chiara F. Bassignana, Irma Wynhoff

**Affiliations:** ^1^ De Vlinderstichting Dutch Butterfly Conservation Wageningen The Netherlands; ^2^ Wildlife Ecology and Conservation Group, Department of Environmental Sciences Wageningen University & Research Wageningen The Netherlands; ^3^ School of Life Sciences, Westville Campus, University of KwaZulu‐Natal Durban South Africa; ^4^ Department of Environmental Science, Faculty of Science Radboud Institute for Biological and Environmental Sciences (RIBES), Radboud University Nijmegen The Netherlands; ^5^ Department of Life Sciences and System Biology University of Turin Turin Italy; ^6^ Present address: University of Gastronomic Sciences Pollenzo CN Italy

**Keywords:** ecological restoration, ecosystem development, *Myrmica* ants, *Sanguisorba officinalis*, wet meadow

## Abstract

In Europe, 50%–70% of former natural grassland area has been destroyed during the past 30 years due to land use changes, losses are expected to increase in the future. Restoration is thought to reverse this situation by creating suitable abiotic conditions. In this paper, we investigate the effects of sod translocation with specific vegetation to facilitate the restoration of a former intensive agricultural field into a wet meadow. First, starting conditions were optimized including modification of the local hydrology, removal of the fertilized topsoil, application of liming, and translocation of fresh clippings as a seed source. The second part aimed at restoring the habitat for the butterfly species *Phengaris (Maculinea) teleius*, one of the species that was especially affected by the loss of wet meadows. This species engages in a complex myrmecophilous relationship with one host plant, *Sanguisorba officinalis*, and one obligate host ant, *Myrmica scabrinodis*. We used sod translocation to create islands of habitat to promote host plant and host ant colonization. After 4 years following the restoration, we observed that plants spread from the transplanted sods to the surroundings. The vegetation composition and structure of the transplanted sods attracted colonization of *Myrmica* ants into the restored areas. Following the increase in vegetation cover and height, *Myrmica* ant colonies further spread into the restored areas. Therefore, sod translocations can be considered an effective restoration method following topsoil removal in the process of restoring wet meadows to provide a starting point for ant colonization and plant dispersion. With these findings, this paper contributes to the evidence‐based restoration of wet meadows on former agricultural fields, including complex interactions between invertebrates and their required ecological relationships.

## Graphical abstract

The habitat of the parasitic threatened butterfly, *Maculinea teleius* was restored by translocation of vegetation sods to enhance colonization of the host ant, *Myrmica scabrinodis*. Sod translocation can be considered an effective restoration method of fen meadows on former intensive agricultural fields.

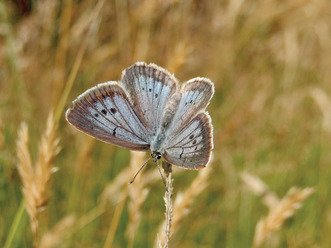

## 1 INTRODUCTION

Landscapes have been severely modified by changes in land use in Europe (Barrett et al., 2018; Newbold et al., 2015; Tscharntke et al., 2012; Warren et al., 2021). Landscapes consisting of a mosaic of natural and seminatural habitat types shaped by traditional low‐intensity agricultural practices have changed into landscapes of large and intensively used agricultural fields or they are encroached by shrubs after abandonment of agriculture (Craioveanu et al., 2021; Loos et al., 2021). In Europe, 50%–70% of former grassland area has been destroyed during the past 30 years (Török et al., 2021). In addition, the highest proportion of habitats with an unfavorable and deteriorating conservation status in the European Union is found in natural grasslands (European Commission, 2015, 2021). European seminatural grasslands supporting a high biological diversity are assumed to have lost at least 90% of their former area during the last century (Cosentino & Schooley, 2018; WallisDeVries et al., 2002). Moreover, climate and land use changes are severe threats to the future of particularly wet grasslands and related species. Landscape homogenization resulted in the fragmentation or loss of habitat for many populations of plant and animal species found in grasslands. The distances between suitable patches have increased and even when grassland habitats are restored, many species are not able to colonize them without support (Bakker & Berendse, 1999). These problems are more evident for species with strict habitat requirements, with a limited distribution, or with low dispersal capabilities (Büchi & Vuilleumier, 2014; Fourcade et al., 2021). Therefore, there is a wide interest in restoring grassland habitats of vulnerable species, however, most restoration projects are based only on vegetation targets or single species while the integration of the whole ecosystem is missing (Goreth et al., 2021; Török et al., 2021).

The first step to restore grasslands on formerly intensively used agricultural fields is the reestablishment of suitable abiotic conditions such as restoring a natural water regime or removing nutrient‐rich top soils (Klimkowska et al., 2007; Zedler & Miller, 2018). Even after solid preparation of the starting conditions, natural colonization of many species to the restored habitats cannot be taken for granted. For certain groups of organisms, translocation offers a possibility for successful colonization into the restored new habitats. For example, plant species have been moved to newly established patches of habitat by transportation of seeds or young plants (Donath et al., 2007; Goreth et al., 2021; Török et al., 2021; Vitt et al., 2016; Wagner et al., 2021), preferably from sites with a common genetic background (Höfner et al., 2021). Moreover, birds, mammals, amphibians, and some butterflies are also translocated as soon as the new patches have developed into suitable habitats (Ferrer et al., 2017; Germano & Bishop, 2009; Wynhoff, 1998). However, within restoration projects, specific taxonomic groups are over‐represented (Donaldson et al., 2016; Kollmann et al., 2016; Martín‐López, 2009) with birds, mammals, and vascular plants being the main target, while invertebrates are underrated (Kollmann et al., 2016). Recently, the necessity of restoring habitats with a broader view, embracing interactions between species, has been stressed, including trophic interactions, as pollination, soil fertility, or bio‐engineers (European Commission, 2021; Fraser et al., 2015; Kollmann et al., 2016). For instance, including interactions between species that have proven to be so‐called ecosystem engineers, such as earthworms or ants could further enhance the success of habitat restoration (Lavelle et al., 2016).

Grassland butterflies are some of the most affected organisms of the changes in natural grasslands (Van Swaay et al., 2015; Warren et al., 2021). This group of insects can be used as indicators of grassland status and effectiveness of applied restoration methods (Musters et al., 2013; Van Swaay et al., 2015). A meta‐analysis of prairie grassland restoration showed that butterfly abundance increased more than bee abundance, especially with multiple restoration methods applied, and older restorations showed the strongest improvements (Sexton & Emery, 2020). However, complex interactions between species such as the case of *Phengaris (Maculinea)* butterflies which have a parasitic relation with ants were not included (Sexton & Emery, 2020). The only example of a successful restoration of these complex host–parasite interactions in butterflies is found in the United Kingdom where limestone grassland habitats of the butterfly *Phengaris (Maculinea) arion* have been restored (Thomas et al., 2009). In 1979, the first reintroductions of *M. arion* started and 30 years later, ca. 40 sites have been colonized by the butterfly thanks to the strong emphasis on the relationship between this butterfly and its local host ant (Thomas et al., 2009). Therefore, restoration projects aiming to improve the conservation status of butterflies with complex host–parasite interactions should have a broader view and focus on their interactions. This paper describes the habitat restoration within the LIFE+ project “Blues in the marshes” for a butterfly species with a comparable life cycle to *M. arion* but restricted to wet fen meadows, where the interactions of invertebrates with the grassland ecosystem is the main focus (Natuurmonumenten, 2018).

**FIGURE 1:**
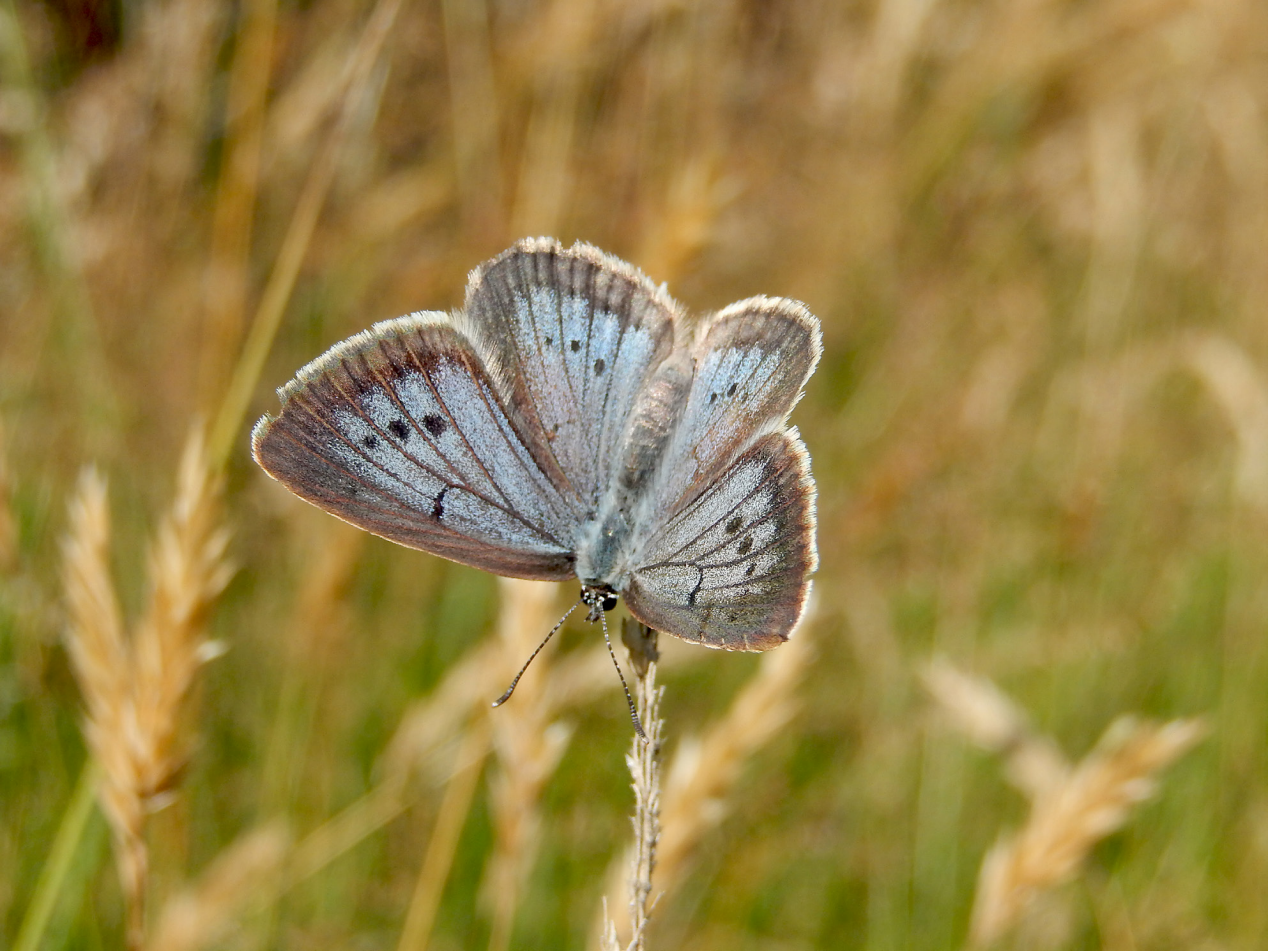
*Maculinea (Phengaris) teleius* myrmecophilous butterfly (Photo: Kars Veling)

The project aims to enlarge the wet meadow habitat of the butterfly species *Phengaris (Maculinea) teleius* (from now on *M. teleius*) (Figure 1) by creating suitable conditions in the surrounding areas for the butterfly population to expand (Natuurmonumenten, 2018). In the Netherlands, only one population of this rare butterfly exists after being reintroduced in 1990 (Wynhoff, 1998), but it has been confined to only 3 ha for more than 25 years. The young caterpillars of the butterfly are monophagous on the host plant *Sanguisorba officinalis*, which is abundant on moist fen meadows (Thomas, 1984). After 3 weeks feeding on the plant, the caterpillar is adopted by the host ants and taken into the ant nest where it hibernates (Witek et al., 2010). There are several host ants for *M. teleius* across Europe (Tartally et al., 2019). However, in the Netherlands, the caterpillars survive only in nests of *Myrmica scabrinodis* and usually on meadows with only this single species present (Van Langevelde & Wynhoff, 2009). Since both host plants and host ants are needed for its survival, the restoration process is necessarily based on the requirements for these two host species to provide suitable habitat for the butterfly. Thus, the major challenge to achieve the restoration of *M. teleius* habitat, is to reach an adequate density of both host plants and host ant nests to enable survival after colonization of the butterfly. Another problem of this system is that both hosts have low propensities to colonize new areas through dispersal (Elmes et al., 1998; Matus et al., 2003). Therefore, in early stages of the restoration, the host plant was translocated with fresh clippings as seed source from nearby wet fen meadow vegetation and with sod translocations (Wynhoff et al., 2017). Sod translocations consist of a transplant of the target vegetation from wet meadows into the restoration area, which are expected to also increase the probability of ant colonization (Wynhoff et al., 2017).

In this study, we investigate the effects of sod translocations of the target vegetation to the restoration areas on the establishment of the host plant *S. officinalis* and the host ant *M. scabrinodis* for the threatened butterfly *M. teleius* over the course of 4 years. We hypothesized that sod translocations accelerate the vegetation development in the restoration area (hypothesis 1). The transplanted sods are expected to promote *M. scabrinodis* colonization and establishment. Our hypothesis was that *Myrmica* ants colonize the restoration areas starting in the translocated sods as these sods are assumed to be islands of suitable habitat for them (hypothesis 2). Over the course of time, the vegetation is expected to get denser and taller, further promoting the distribution of *M. scabrinodis* in the restoration areas (hypothesis 3).

## 2 METHODS

### 2.1 Study site

**FIGURE 2:**
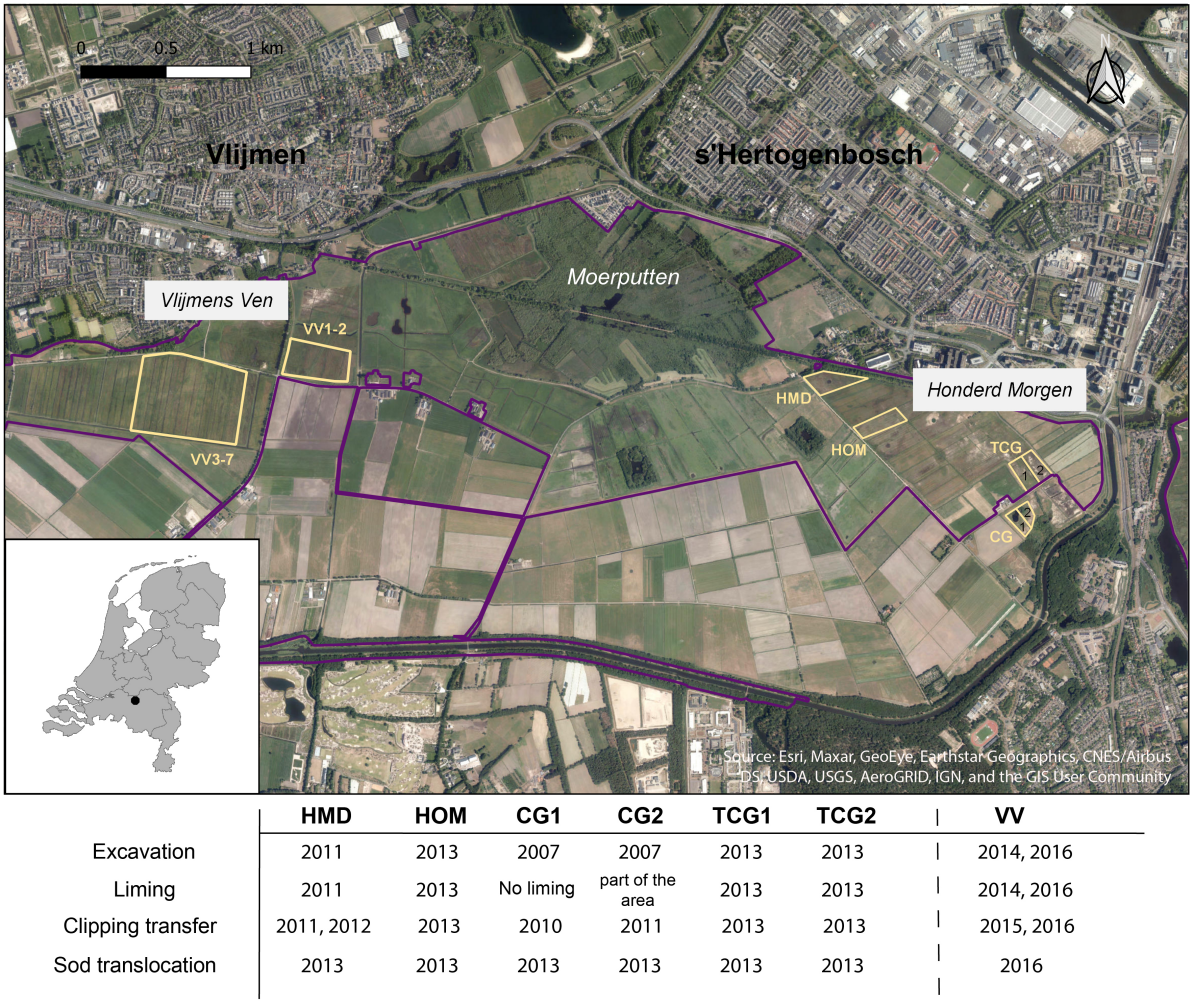
Study area. Natura 2000 nature reserve (purple line) with the core area of Moerputten and restored areas, Vlijmens Ven (VV) and Honderd Morgen (The Netherlands). Yellow polygons locate restored patches: VV1 to VV2, VV3 to VV7, HMD (=Honderdmorgensedijk Driehoekje), HOM (=Honderdmorgensedijk), TCG1 and TCG2 (=Tegenover Compensatiegebied) and CG1 and CG2 (=Compensatiegebied). The table below shows the restoration methods applied in different years during the research.

A restoration project was carried out in the Natura 2000 area “Vlijmens Ven, Moerputten and Bossche Broek” (931 ha), located south of the city of 's Hertogenbosch, the Netherlands (Figure 2). The core site Moerputten (115 ha) consists of moist meadows and wet forests. In the past, the surrounding area was dominated by intensively used agricultural fields and cattle pastures (Wynhoff, 1998; Wynhoff et al., 2017). The wet meadows in Moerputten provide the habitat for *M. teleius* which is restricted to one core population in this reserve. The restoration actions were described in detail earlier (Wynhoff et al., 2017). The restoration areas were at distances from the butterfly population within the known long dispersal range (average 2 km, maximum 4.5 km) (Van Langevelde & Wynhoff, 2009). In the restoration areas, suitable abiotic conditions were restored in terms of basic seepage, water accessibility, removal of the fertilized soil, and the preservation of high winter water tables to maintain nutrient‐poor conditions (Wynhoff et al., 2017). The top 40 cm of phosphate‐enriched soil on a total of 250 ha was excavated. The development of the target vegetation was facilitated by liming (1000 kg/ha) and transfer of freshly cut clippings on the excavated areas from the nearby nature reserve (Donath et al., 2007; Höfner et al., 2021; Hölzel & Otte, 2003; Matus et al., 2003; Török et al., 2011). Starting 1 year later, all restored meadows were mown yearly in summer. Finally, vegetation sods consisting of a transplant of suitable habitat for *M. teleius* were translocated from meadows in Moerputten (see details below and in Figure 2).

### 2.2 Sod translocation experiment

The sod translocation experiment was conducted twice, the first one in 2013 consisted of six patches in *Honderd Morgen* area (Figure 2) and the second in 2016 consisted of seven patches in *Vlijmens* Ven area (Figure 2). On 23 October 2013, the first sod translocation was carried out in *Honderd Morgen* area at four locations (two patches in CG and two in TCG, one patch in HOM, and one in HMD; Figure 2). We translocated 54 sods (1.25 × 0.85 m each, 10 cm thick) from three fen meadows in Moerputten nature reserve where the vegetation had been mown 1 week before. Each sod was first marked and separated from its surrounding by cutting the edges (Figure S1a). Then, the 10‐cm‐thick top layer was separated from the underground with a dense prong to avoid tearing (Figure S1b,c). The sods were placed on plastic road plates for transportation (Figure S1d,e) and carefully placed into an earlier dug out ditch in the restoration area (Figure S1f). Three years later, on 4 October 2016, the second series of sods was translocated to *Vlijmens* Ven (VV). One week after mowing, 63 sods of the same size as in 2013 were removed from the nature reserve and spread over seven different locations in VV (Figure 2; for detailed information watch the video Figure S1h).

As a consequence of the weather conditions in both years of sod translocation (average temperature in both years of 11°C following a week of cold weather), *Myrmica* queens were assumed to hide deep in the soil (>10 cm deep) for hibernation (Kipyatkov & Lopatina, 1999) and hence not be translocated together with the sods. To test whether this assumption is true, we sampled ants three times during the second sod translocation in October 2016. The first time was right after lifting the sods, and we found worker ants under seven sods. Then we placed ant baits in the translocated sods after 1 week and after 10 months of the translocation, and we found worker ants in seven and five sods, respectively (I. Wynhoff, unpublished data). In only one sod, worker ants were found more than once and the rest of the captured ants were distributed randomly in each capture event. Thus, since we also did not find queens, we concluded that effectively ants were not translocated within the sods and that those found during the experiment (e.g., in 2014; Wynhoff et al., 2017) were colonizing from outside the topsoil removed area.

**FIGURE 3:**
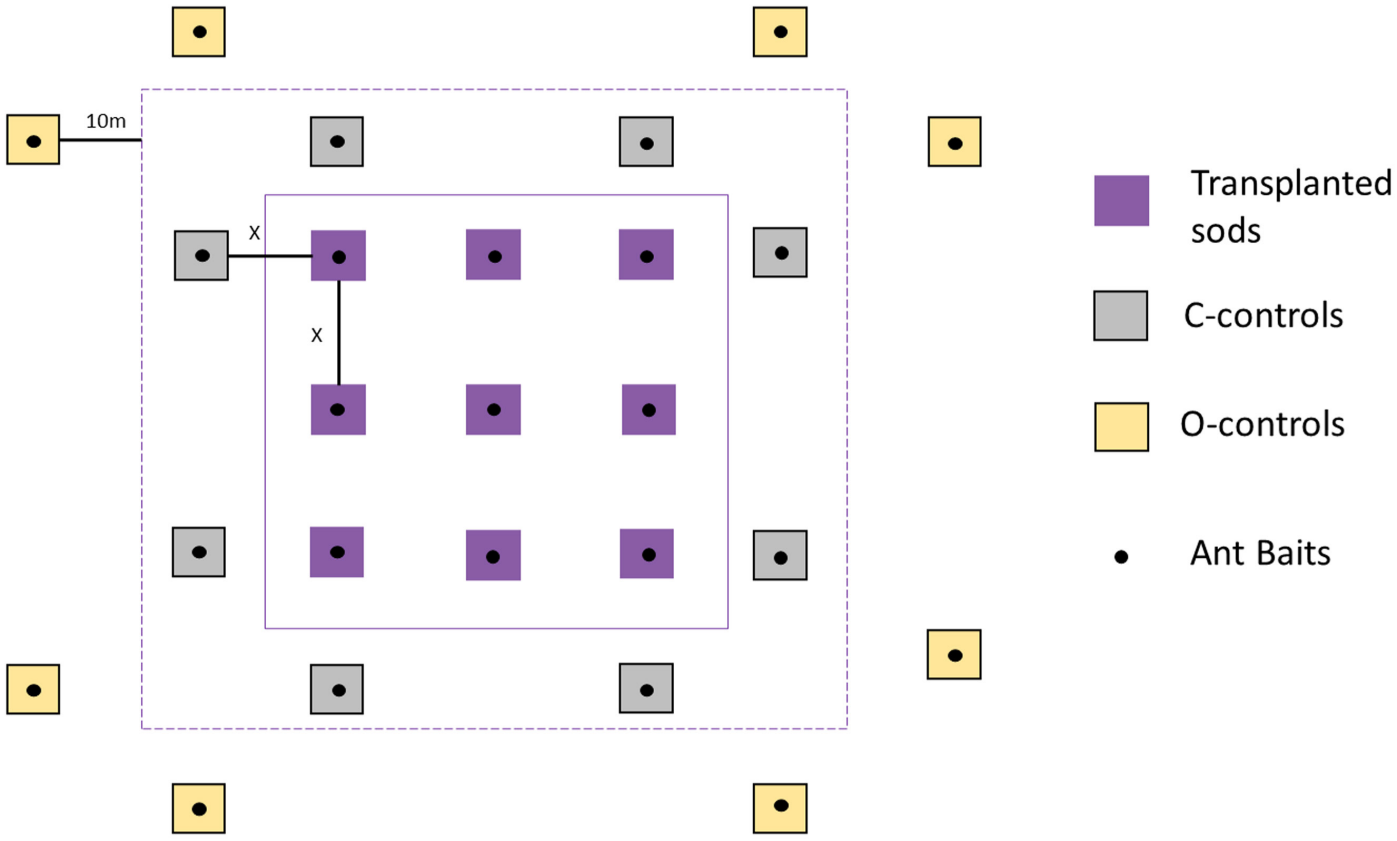
Location of the nine transplanted sods (purple) and control relevés (c‐controls: grey) within a patch. C‐controls were placed randomly. Distance between the sods and c‐controls (x) is 3 m for Honderd Morgen meadows and 6 m for Vlijmens Ven meadows. Black dots indicate ant baits. In 2015, an additional 8 controls were placed outside each patch at a distance of at least 10 m (o‐controls: yellow).

In our study, sods were moved to sandy soil with sparse vegetation in different densities. At each patch, nine sods were placed in a 3 × 3 grid (Figure 3, Figure S1g). In 2013, a distance of three meters was kept between the sods. Control plots of the same dimensions of the sods were established at the same distances around the sods (c‐controls in Figure 3). It is expected that worker ants from the same colony could only be found in one sod or control; thus, the frequency of ant occurrences within a patch was assumed to be independent of species' activity densities (Dahms et al., 2010). After the rapid colonization of the sods in 2014 (Wynhoff et al., 2017), eight additional controls per patch were added at random locations of at least 10 m distance from the patch in 2015 (o‐controls in Figure 3). The second translocation in 2016 (VV meadows) mainly serves to prove that effectively ants were not translocated with the sods, therefore, the second translocation data are not included in the analysis. All meadows with translocated sods were managed equally. In each year after sod translocation, they were mown in October/November. After cutting, the hay was left there for several days and then removed.

### 2.3 Data collection

In July and August 2014 and 2016, 1 m^2^ vegetation relevés were performed on the sods and the c‐controls of the first sod translocation experiment in 2013 according to the Braun Blanquet method (Meijden & Bruinsma, 2007). All plant species were listed and their coverage was estimated. The Ellenberg values of nitrogen, moisture, and pH per relevé were calculated using the program Turboveg (Hennekens & Schaminée, 2001). Every year from 2014 until 2017, the vegetation structure was recorded including the cover of shrubs, herbs, mosses, total vegetation, dead organic matter (from now on DOM), and bare soil on all transplanted sods and controls. In addition, we measured the height of the vegetation using the Barkman stick method (Barkman, 1979; Wynhoff et al., 2017). In total, five measurements were taken per relevé and were averaged. The standard deviation (SD) was used as a proxy for variation in vegetation structure.

To collect data on ant presence in all patches, plastic pitfall tubes were placed (15 ml, Ø1.7 cm, 12 cm long) filled with fruit wine (mixture of raspberry, blackcurrant, cherry, 8.5% alcohol) in the soil in the middle of the plots, with the top of the tube level with the ground surface. Tubes were collected 24 h after positioning, covering all periods of daily activity of the ants. Baits were placed between mid‐July and August every year. All ant species were identified using Boer (2010).

### 2.4 Data analysis

#### 2.4.1 Vegetation

First, differences in vegetation structure were analyzed to assess the effect of year of experiment and treatment (sods and c‐controls for the 4 years of research and o‐controls for the last 3 years) on different environmental variables that experienced changes. We performed beta regression models with a Beta distribution for the variables measured as a percentage (i.e., total vegetation cover, shrub cover, DOM cover, moss cover, and *Sanguisorba* cover), and GLMMs with a normal distribution for height variables (i.e., mean and Standard Deviation vegetation height) using Patch ID as random factor. For the beta regression models, we calculated the significance of each environmental variable by using a likelihood ratio test between models with and without a specific environmental variable. We used the package betareg (Cribari‐Neto & Zeileis, 2010) for the beta regression analysis, version 5.3–4, and package lme4 version 1.1.21 (Bates et al., 2015) for the GLMM, using the software R, version 3.6.3 (R Core Team, 2021).

The changes in the vegetation composition on the sods and the c‐controls between 2014 and 2016 were investigated using a multivariate statistical analysis, Detrended Correspondence Analysis (DCA) from the package vegan, version 2.5–7 (Oksanen et al., 2020). All environmental variables were included in the ordination. Spearman correlations were performed between each environmental variable and the scores of the first DCA axis. A *t* test analysis was performed to detect differences between DCA axes coordinates for the relevés in the two different years of experiment (2014 and 2016), both variables had a normal distribution.

#### 2.4.2 Ants

To test whether ants colonized the restoration areas starting in the translocated sods, we tested whether differences in the presence/absence of *M. scabrinodis*, *Lasius niger*, all *Myrmica* species and all ant species together were determined by the treatment, year of experiment and their interaction with a series of Generalized Linear Mixed Model (one GLMM analysis was done for each). In these GLMMs, we used a binomial distribution with logit link function and Patch ID as random factor.

In addition, another series of GLMM (with binomial distribution and logit link function) was performed to test which environmental variables affect the occurrence of ants in the restoration areas; we incorporated all environmental variables in the GLMMs (one factor included in each model) for the presence/absence of the ants. Environmental variables were standardized to compare the effect sizes of them. Here we used year of experiment as random effect, the highest estimated value of the coefficients to determine which variable explained ant presence best and the adjusted *p*‐values according to Benjamini‐Hochberg procedure using a false discovery rate of 10% for significant values (FDR = 0.1) (Benjamini & Hochberg, 1995). Additionally, to test the presence of *L. niger* on the establishment success of the *Myrmica* ant species, we included the presence of *L. niger* as an independent variable in the *M. scabrinodis* model and, in the opposite way for the model of *L. niger*.

Finally, we calculated the predicted probabilities of encountering *M. scabrinodis* and *L. niger* along the gradient of significant environmental factors for *M. scabrinodis* (i.e., total cover of vegetation, bare soil cover, and vegetation height). For the predicted probabilities, we decided to not include the year of experiment as a random factor to see the general trend representing the average effect over the years and either, the herb cover for being correlated with total vegetation cover.

## 3 RESULTS

### 3.1 Vegetation

All sods survived the transplantations of 2013 and subsequent years of the research. After the start of the experiment, the vegetation structure of all plots changed during the years of experiment and differences between treatments and locations were found as a consequence of vegetation development. We found significant differences in total vegetation cover, herb cover, shrub cover, bare soil cover, DOM cover, moss cover, mean, and SD of vegetation height, between the years of experiment and treatments, and *Sanguisorba* cover only between treatments (Table 1). Overall, the sods showed a higher total vegetation cover, higher vegetation height and more *Sanguisorba* plants over the years in comparison with the c‐controls and the o‐controls (Table S2). In the last year of the experiment (2017), all sods were almost fully covered by vegetation while the control plots had more bare soil (Figure S3). Moreover, the host plant of *M. teleius*, *S. officinalis*, was found mostly in the sods compared to the controls (Figure S3).

**TABLE 1:** Results of the series of models for environmental variables that experience changes on the influence of year and treatment

Beta regression model	*Χ* ^2^	df1	df2	*p*‐value
Total vegetation cover				
Year of experiment	28.4	3		<.001**
Treatment	236.2	2		<.001**
Herb cover				
Year of experiment	81.2	3		<.001**
Treatment	440.2	2		<.001**
Shrub cover				
Year of experiment	8.5	3		.038*
Treatment	75.1	2		<.001**
Bare soil cover				
Year of experiment	13.2	3		.004*
Treatment	228.4	2		<.001**
DOM cover				
Year of experiment	186.5	3		<.001**
Treatment	29.5	2		<.001**
Moss cover				
Year of experiment	32.4	3		<.001**
Treatment	158.1	2		<.001**
Sanguisorba cover				
Year of experiment	0.07	3		.965
Treatment	69.3	2		<.001**

GLMM model	*F*	df1	df2	*p*‐value
Mean vegetation height				
Year of experiment	53.15	3	552	<.001**
Treatment	163.01	2	552	<.001**
SD vegetation height				
Year of experiment	43.85	3	552	<.001**
Treatment	13.79	2	552	<.001**

*Note*: Year of experiment and treatment were included as independent variables. Values of the table presented in the columns: Coefficient *Χ* ^2^, coefficient F (F), degrees of freedom 1 (df1), degrees of freedom 2 (df2) and *p*‐value (*<.05, **<.001).

**FIGURE 4:**
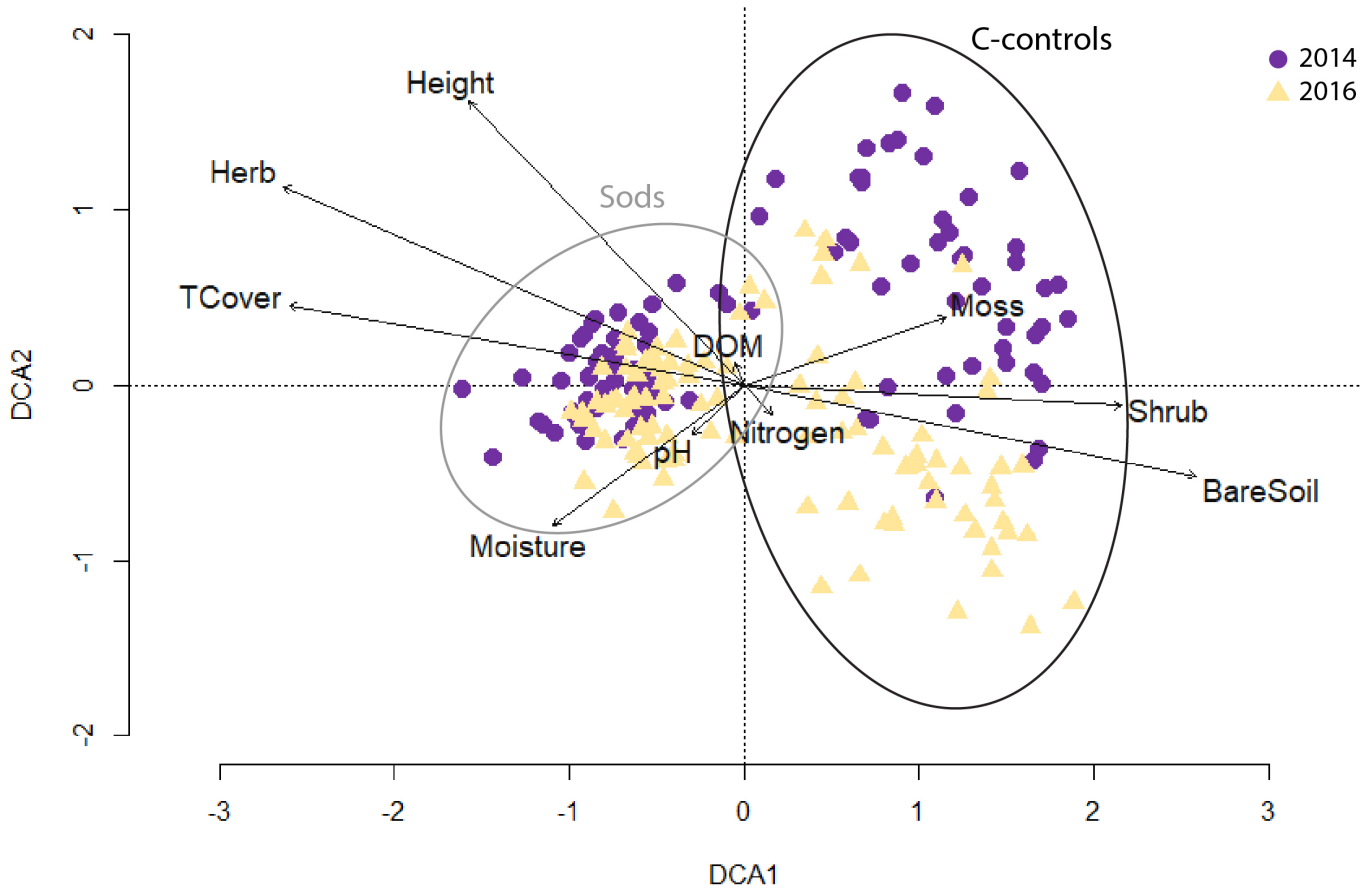
DCA ordination of the vegetation relevés of the restored areas comparing 2014 and 2016. Purple circles are plots in 2014 and yellow triangles in 2016; big grey circle assembles the sods and big black circle groups the c‐control plots in both years. Environmental factors are represented with arrows expressing their gradient with the length. Nitrogen, pH and Moisture refer to the respective Ellenberg indicator values calculated from the relevés. Herb, Moss, BareSoil, TCover, Shrub and DOM refer to the cover (in %) of herbs, mosses, bare soil, total vegetation, shrubs and dead organic matter respectively.

The DCA showed differences in plant species composition between 2014 and 2016 (Figure 4). In total, 100 species were found in 2014 and 51 new plant species were detected in 2016. The first DCA axis divided the relevés into two groups. The sods were clustered on the left‐hand side, influenced by several environmental variables correlated with the first DCA axis: herb cover (Pearson correlation coefficient ρ = −.74, *p* < .001, df = 178), vegetation height (ρ = −.60, *p* < .001, df = 178), Ellenberg moisture value (ρ = −.36, *p* < .001, df = 178), and total vegetation cover (ρ = −.67, *p* < .001, df = 178). On the right‐hand side, the c‐controls are more scattered due to the lack of similarity between plots (Figure 4). The c‐control plots were correlated with moss cover (ρ = .27, *p* < .001, df = 178), shrub cover (ρ = .58, *p* < .001, df = 178), and bare soil cover (ρ = .66, *p* < .001, df = 178). The second DCA axis separated the plots between the years of experiment with the 2014 plots in the upper part of the graph and the 2016 plots in the bottom part (Figure 4). The vegetation development shows a transition toward increasing values of Ellenberg indicators for pH, nitrogen, and moisture. The DCA analysis of the vegetation in 2014 and 2016 presents an eigenvalue of 0.46 for the first axis and 0.24 for the second axis. Regarding the DCA1 axis scores, no difference between the years of experiment was found. However, the analysis of the DCA2 axis scores showed a significant difference between 2014 and 2016 (*t* test, *t* = 10.70, df = 101, *p* < .001), corroborating the significant shift of species composition between just 2 years of development.

### 3.2 Ants

**FIGURE 5:**
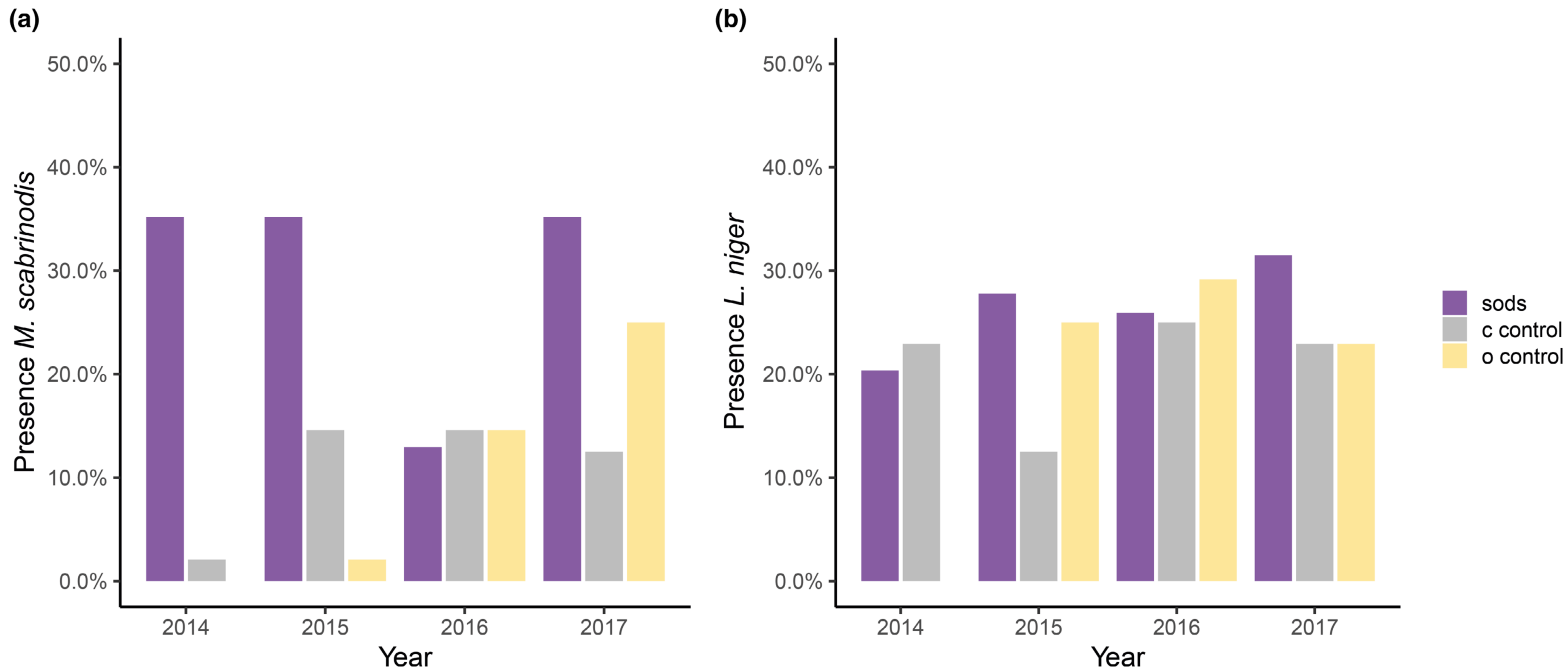
Percentage of presence of (a) *Myrmica scabrinodis* and (b) *Lasius niger* across the plots of the different treatments (sods, c‐controls and o‐controls) along the years of experiment.

Ten ant species were captured throughout the four investigated years, but only four species were found every year: *L. niger*, *M. scabrinodis*, *Myrmica sabuleti*, and *Myrmica gallienii*. *M. scabrinodis* and *L. niger* appeared every year in all treatments, whereas *M. sabuleti* and *M. gallienii* were sampled every year at least in one sod and in some controls (Table S4). The effect of treatment and year of experiment affected the investigated ant species or groups differently (Table 2). Only *M. scabrinodis* occurrence significantly fluctuated over time while the presence of *L. niger* was affected by neither treatment nor year of experiment (Table 2, Figure 5). *M. scabrinodis* was mostly present in the sods compared to the controls over the years, while *L. niger* was evenly found in all treatments (Figure 5). The colonization of *M. scabrinodis* started in 2014 from the sods to the controls through the years (Figure 5 and Figure S5a). However, in 2016 there was a decrease in its presence in the sods due to heavy rains and short periods of flooding during spring and summer.

**TABLE 2:** Results of the four generalized linear mixed models on the influence of year of experiment and treatment on the occurrence of the ants

Model	F	df1	df2	*p*‐value
*Myrmica scabrinodis*				
Year of experiment	3.24	3	550	.02*
Treatment	7.42	2	550	<.001**
Year ex. × treatment	3.31	5	550	.006*
*Lasius niger*				
Year of experiment	0.99	3	550	.39
Treatment	1.72	2	550	.18
Year ex. × treatment	1.32	5	550	.25
*Myrmica* species				
Year of experiment	2.00	3	550	.11
Treatment	14.81	2	550	<.001**
Year ex. × treatment	3.08	5	550	.009*
All ant species				
Year of experiment	1.79	3	550	.12
Treatment	20.24	2	550	<.001**
Year ex. × treatment	2.32	5	550	.05

*Note*: Year of experiment, treatment and interaction between them were included as independent variables. Values of the table present in the columns: Coefficient F (F), degrees of freedom 1 (df1), degrees of freedom 2 (df2) and *p*‐value (*<.05, **<.001).

The effect of treatments was significant for the presence of *M. scabrinodis*, all *Myrmica* species, and all ant species. For *M. scabrinodis* alone and all *Myrmica* species, we found a significant interaction between the years of experiment and the treatments (Table 2). The presence/absence of all ant species found on the investigated plots was different between the treatments but not for the years of experiment since sods translocation (Table 2, Figure S5b).

**FIGURE 6:**
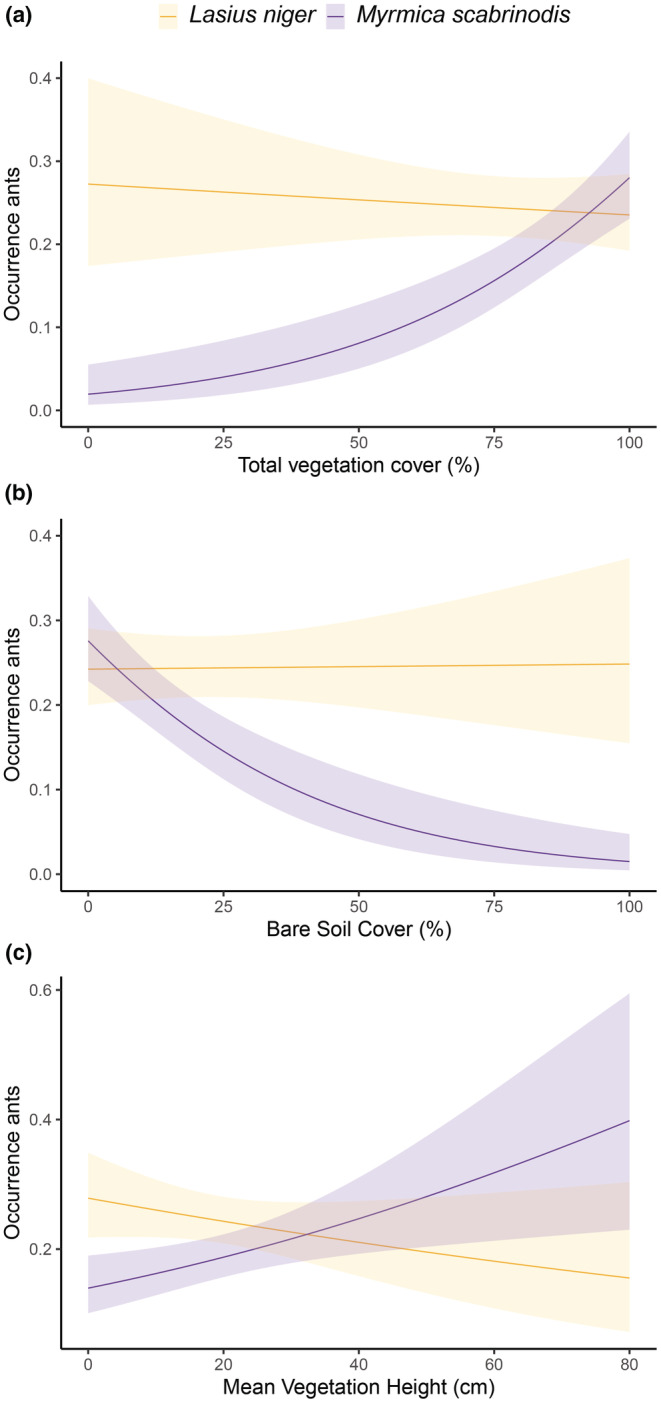
Predicted probabilities (±CI) of occurrence of *Myrmica scabrinodis* and *Lasius niger* ants as a function of (a) total vegetation cover, (b) bare soil cover, and (c) mean vegetation height.

We found effects of the changes in certain vegetation parameters on the ants (Table 3). The distribution and presence of *M. scabrinodis* were mostly correlated with the environmental variable total vegetation cover and bare soil cover showing the higher effect sizes resulting in higher probabilities of occurrence (Table 3, Figure 6a,b). These last two variables have opposite effects and were highly correlated (Pearson correlation, ρ = −.85, *p* < .001**, Figure S6). As the herb cover was correlated with the total vegetation cover (Pearson correlation, ρ = .87, *p* < .001**), it also showed a large impact on the presence of this ant. The mean vegetation height also was significant but its effect size was lower, with *M. scabrinodis* being more likely to occur in areas with taller vegetation (Figure 6c). The cover of *Sanguisorba* was just significant for the presence of *M. scabrinodis*. Ellenberg values of nitrogen, moisture, and pH did not have an influence on the presence of *M. scabrinodis*, however those values were significant for the presence of *L. niger* (Table 3). *M. scabrinodis* avoided areas where *L. niger* was present, suggesting a competition effect between both ant species. The year of excavation was significant for the presence of the ants; the more years passed since excavation the higher the probability of finding ants. On the other hand, the presence of *L. niger* was correlated to fewer and different variables (Table 3). The negative correlation with *M. scabrinodis* showed the largest impact. Shrub cover had a significant effect on the occurrence of *L. niger*. Ellenberg values had a negative effect on the presence of *L. niger* where nitrogen had a high impact. The probability of *L. niger* 's occurrence slightly decreased with increasing total vegetation cover and decreased drastically with higher vegetation (Figure 6a,c), but was not affected by bare soil cover (Figure 6b). Variables benefitting *M. scabrinodis* showed a negative effect on *L. niger*.

**TABLE 3:** Generalized linear mixed model result of *Myrmica scabrinodis´* and *Lasius niger’* presence based on various influential factors after the sod translocation in 2013

	Model	*Myrmica scabrinodis*					Model	*Lasius niger*				
		AIC	Estimate	SE	*z*	Adjusted *p*‐value		AIC	Estimate	SE	*z*	Adjusted *p*‐value
*N* = 550	Bare soil cover	505	−0.89	0.18	−4.93	.014*	Year of excavation^a^	320.8	–	0.16	–	.007*
	Total veg. cover	507.6	0.83	0.17	4.91	.021*	Shrub cover	583.7	0.53	0.09	5.6	.014*
	Herb cover	510.6	0.68	0.13	5.11	.007*	*Myrmica scabrinodis*	611.7	−0.61	0.29	−2.14	.043*
	Year of excavation^a^	525.1	–	0.24	–	0.029*	Herb cover	613.5	−0.17	0.09	−1.82	.050
	Mean height	535.1	0.28	0.11	2.61	.036*	Moss cover	614.4	−0.15	0.10	−1.50	.057
	*Lasius niger*	536.8	−0.61	0.29	−2.15	.043*	Mean height	615.0	−0.13	0.10	−1.32	.064
	Shrub cover	537.7	−0.25	0.13	−1.91	.057	DOM cover	616.2	0.07	0.09	0.77	.071
	Moss cover	540.2	0.14	0.1	1.3	.064	Stdev height	616.3	0.06	0.09	0.69	.079
	DOM cover	541.3	0.08	0.11	0.76	.079	Total veg. cover	616.5	−0.05	0.09	−0.54	.086
	Stdev height	541.7	0.05	0.11	0.46	.086	Bare soil cover	616.8	0.008	0.09	0.09	.100
*N* = 502	Ellenberg moisture	515.5	0.14	0.11	1.24	.071	Ellenberg nitrogen	536.0	−0.6	0.12	−4.84	.021*
	Ellenberg pH	517	0.03	0.11	0.34	.093	Ellenberg moisture	553.4	−0.31	0.10	−3.06	.029*
	Ellenberg nitrogen	517.1	0.0008	0.11	0.01	.100	Ellenberg pH	554.7	−0.29	0.10	2.82	.036*
*N* = 448	*Sanguisorba* cover	436.8	0.21	0.11	1.99	.050*	*Sanguisorba* cover	509.7	0.04	0.10	0.44	.093

*Note*: Each row refers to one independent variable (one model per row) for the specific ant presence. In the columns appear AICs, estimate, StE (standard error), *z* (*z* value), and adjusted *p*‐value. All variables have 1 degree of freedom. *p*‐values* equal or less than 0.05. Adjusted *p*‐values according to Benjamini‐Hochberg procedure. Cover variables have *N* = 550 except Ellenberg indicator values, *N* = 502 and *Sanguisorba* cover, *N* = 448.

^a^ Categorical variable.

* Significant values (FDR = 0.1).

The results of the GLMM for all *Myrmica* ant species presence (Table S7) showed that the presence of *L. niger* is the most influential variable with the higher effect size. Similarly, to the case of *M. scabrinodis*, higher cover of bare soil decreased *Myrmica* occurrence while herb cover and total vegetation cover increased *Myrmica* occurrence. The cover of *Sanguisorba* also increases *Myrmica* presence significantly. The Ellenberg moisture value, year of excavation, mean height, and shrub cover also have a significant influence on the presence of all *Myrmica* species, however their effect size is low. Finally, the occurrence of all ant species is mostly influenced by Ellenberg nitrogen value, followed by bare soil cover, total vegetation cover, *Sanguisorba* cover, and herb cover (Table S7).

## 4 DISCUSSION

The loss of wet meadows has been dramatic in Europe during the last century, and nowadays, they are still threatened by climate and land use changes (Cosentino & Schooley, 2018; Joyce, 2014; WallisDeVries et al., 2002). In this paper, we investigated whether the main requirements of *Maculinea teleius* butterflies as inhabitants of fen meadows can be restored after top soil removal and sod translocation. Restoration began with expanding the range of wet meadow conditions and plants, followed by ant colonization and dispersal.

Four years after translocating vegetation sods of wet meadows into barely vegetated restoration areas, the composition and structure of the vegetation slightly changed and the sods remain stable and without deterioration, improving the status of the meadows with respect to *Myrmica* ant colonization (Table 2, Figure 5). Even though clippings from fen meadow vegetation had been spread before the translocation experiment took place, vegetation development has not been fast enough to catch up with the mature status of fen meadows' vegetation. Sod characteristics reflect a long history of vegetation development that has taken place over many years; they have a high coverage of vegetation, reflecting the future development of the control plots (Table 1, Figure 4). Controls around sods have had only little time for plant colonization and vegetation establishment, showing environmental characteristics from primary phases as lack of vegetation coverage, more moss cover, and high levels of nutrients (Smith et al., 2002; Zedler, 2000).

Here, we showed how the restoration area (sods) acquired already wet meadow characteristics over the study period, and the vegetation composition on the c‐controls may shift toward the vegetation composition of the sods (Figure 4). Sod translocation leading to the simple proximity of the target vegetation community might help plant propagation and increase the likelihood of success to cover the area over time (Jansen et al., 2000; Matus et al., 2003). Indeed, a remarkable number of 51 new species was found in a short time, determining an important difference between the years of experiment. One of the plants benefitting from the restoration was the host plant *Sanguisorba officinalis*, which colonized the whole restoration area (personal observations) after spreading of clippings and seeds and translocation of sods. Though the spreading of clippings has been important to start the target vegetation growing, the translocated sods were added to improve the colonization of the desired vegetation, supporting hypothesis 1. An increase in the vegetation cover triggers other environmental conditions important for the restoration; when the vegetation covers more ground, more humidity is captured in the topsoil and under the herb layer which creates a favorable microclimate for many insect species (Procházka et al., 2011). The slight (but nonsignificant) increase of the Ellenberg moisture values in the restoration area points into that direction (Figure 4, Table S2). Moreover, the accumulation of dead organic matter (DOM) is one consequence derived from vegetation development toward more mature ecosystem stages (Jansen et al., 1996). DOM interacts with several conditions of the soil, physically and chemically, that link the soil biodiversity and ecosystem functions (Bot & Benites, 2005). With our restoration, DOM fluctuated over time, increasing only for c‐controls (Table 1 and Table S2). Finally, the reduction of moss cover in the sods and c‐controls, slight decrease of Ellenberg nitrogen value in sods and c‐controls and the appearance of higher shrub coverage in the c‐controls and (nonsignificant) in the o‐controls are also signs of the development shifting away from primary phases over time (Middleton, 2018). In our experiment, the o‐controls performed better than the c‐controls. All these characteristics that we observed during the study period, indicate that the vegetation structure is moving toward a healthy wet meadow (Sammul et al., 2012; Zedler, 2000).

Our results showed that the sod translocation method enabled the host *Myrmica* ants to colonize new areas where they were initially absent due to the soil excavation. We validated that no queens were moved with the sod translocation, so colonization was dependent on external founders: young mated queens dispersing after their nuptial flights. The ants found in the sods came from outside the topsoil removed areas. *M. scabrinodis* generally avoids areas dominated by bare soil because the conditions are extreme, with high temperatures and drought during summer days, while moist conditions and moderate temperatures are kept stable by vegetation cover (Elmes et al., 1998; Trigos‐Peral et al., 2018; Wynhoff et al., 2017). Therefore, if the vegetation cover around the sods was increasing, the ants could occupy those areas that offered their required ecological conditions of moisture and temperature (Elmes & Wardlaw, 1982; Procházka et al., 2011). Indeed, the different restoration methods applied, and in particular the sod translocations, allowed a fast colonization of *M. scabrinodis* (Figure 5). These findings support our hypothesis 2. Only in 2016, the process was slowed down due to frequent heavy rains in the summer. As the area covered by vegetation as well as its height increased, the probability of occurrence of *M. scabrinodis* also increased (Figure 6), supporting our hypothesis 3. Furthermore, to be able to build a nest, *Myrmica* ants need some support by plant material, such as roots or stems but *L. niger* is able to start a colony in a shallow nest without any support (Kipyatkov & Lopatina, 1999). While the vegetation outside the sods grew and increasingly resembled that of the sods, the *Myrmica* ants progressively invaded the area around the sods (Figure 5). Plots outside the worker activity range of the sod nests were colonized as well suggesting young mated queens during their nuptial flights were attracted by the vegetation structure characteristics. In our study *M. scabrinodis* has been found in the surroundings of *S. officinalis*, close to the stem base of the plants. Females of *M. teleius* have been shown to lay their eggs on selected host plants that are surrounded by *Myrmica* nests (Wynhoff et al., 2008; Wynhoff & van Langevelde, 2017). This choice increases the probability of the caterpillars to proceed their development in the ant nest. Our data shows that *S. officinalis* cover increases the presence of *M. scabrinodis* but this is because both butterfly hosts, *M. scabrinodis* and *S. officinalis*, have similar ecological requirements. Therefore, in the coming years the probability is high that they will continue to occur in each other's vicinity.

In the restored meadows, spatial separation of the two main ant species was found. The presence of bare soil negatively influences the presence of *M. scabrinodis* and facilitates the colonization and spreading of its main competitor, *L. niger* (Figure 6). During colonization of new habitat, the presence of *L. niger* may obstruct the colonization of *M. scabrinodis* (Elmes et al., 1998). Indeed, we found a negative correlation between the two species, which suggests that the species exclude each other (Table 3). Higher presence of the main competitor was detected in areas where the time between the soil removal and the translocation of clippings and sods was longer. In these places, *L. niger* was more dominant than in the other locations which might explain the low values for *M. scabrinodis*. In contrast, in the areas where the restoration interventions occurred in the same year, the presence of *L. niger* was limited or the species was even absent. Sod translocations in areas without *L. niger* provided better starting conditions for the colonization and dispersal of *M. scabrinodis*. This information was carefully taken into account for designing the second set of sod transplantations in 2016 as it was performed directly after the topsoil removal, thus demonstrating the importance of the learning process during a restoration project to restore a complex ecological system such as the one of *Myrmica scabrinodis* and *Maculinea teleius*.

In our experiment, we demonstrated how involving different levels of a complex ecological system improves the success of habitat restoration. As a first step, wetland conditions (hydrology and poor nutrients) were restored and the application of fresh clippings from meadows with the target vegetation helped the target plant species to easily colonize the restored areas. For wet meadows, additionally sod translocation can be a successful method accelerating vegetation development within a short period of time. Though removing the sods from the source meadows leads to partial damage, if it is done carefully the vegetation in the source meadows can cover the gaps quite fast while the start in the restoration meadows is facilitated significantly. The vegetation of the restored meadows got denser and taller, inside and outside of the sods, influencing several environmental variables and it created suitable habitat for the ants to spread. The transplanted sods act as habitat islands attracting *Myrmica* ants. Even within the limited number of years after restoration, we showed that sod translocation can be applied to facilitate wet meadow restoration. The subsequent dispersion of the host plant *Sanguisorba officinalis* and the host ant *Myrmica scabrinodis* can provide new habitat for *Maculinea teleius*. It is helpful if the distance between the new restored habitat and existing populations of the butterfly is within the dispersal potential of the butterfly to allow natural colonization once the ecological requirements of the butterflies are realized on the restored parcels.

In the summer of 2021, 8 years after sod translocation, for the first time, a small population of the *Maculinea teleius* butterflies was found on one of the restored meadows. Frequent transect counts allowed for the calculation of a total population size of 25 ± 3 individuals (I. Wynhoff, unpublished data). Some females must have colonized the meadow 1 year before, accepted the status of the restoration area, and deposited their eggs on the available *Sanguisorba officinalis* plants where the nests of *Myrmica scabrinodis* were large enough to raise the caterpillars. This new population of butterflies proves how the restoration methods described in this paper were successful for them.

## AUTHOR CONTRIBUTIONS

**Cristina G. Sevilleja:** Formal analysis (lead); methodology (equal); project administration (equal); software (equal); writing – original draft (lead); writing – review and editing (equal). **Frank van Langevelde:** Formal analysis (supporting); software (supporting); supervision (equal); writing – original draft (equal); writing – review and editing (equal). **Juan Gallego‐Zamorano:** Formal analysis (equal); software (equal); writing – review and editing (equal). **Chiara F. Bassignana:** Methodology (supporting); writing – original draft (equal). **Irma Wynhoff:** Conceptualization (lead); data curation (lead); funding acquisition (lead); methodology (equal); project administration (lead); resources (equal); supervision (equal); writing – original draft (equal); writing – review and editing (equal).

## CONFLICT OF INTEREST

The authors have no conflicts of interest to declare that are relevant to the content of this article.

## Supporting information


Appendix S1

## Data Availability

Data supporting this article are uploaded at the Dryad repository: https://doi.org/10.5061/dryad.1jwstqjz2.
