# Supplementary material for: Sod translocation to restore habitats of the myrmecophilous butterfly Phengaris (Maculinea) teleius on former agricultural fields
Source: Ecol Evol. 2022 Sep 9;12(9):e9293. doi: 10.1002/ece3.9293 (PMC9463040; doi:10.1002/ece3.9293)
Supplement: Supplementary file 1 — Appendix S1 [file ECE3-12-e9293-s001.docx]

# **Supporting Information S1**. Translocation of vegetation sods as performed in the LIFE+ project “Blues in the marshes”. Pictures from Kars Veling.

S1.A: Marking the sods and cutting the edges.


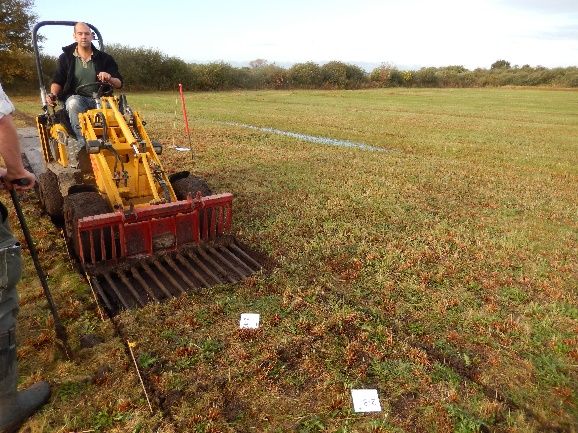


S1.B: Separating the top layer from the underground with a dense prong.


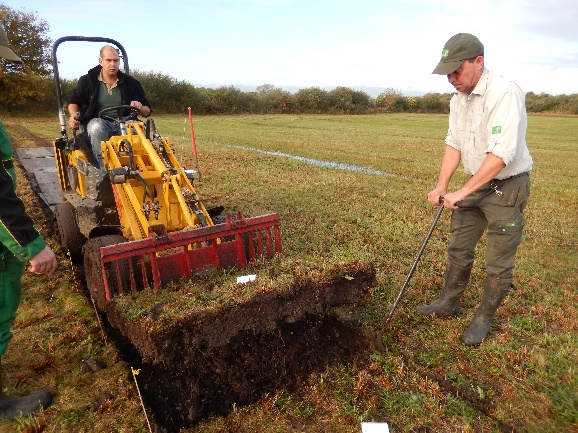


S1.C: Lifting the sod from the underground.


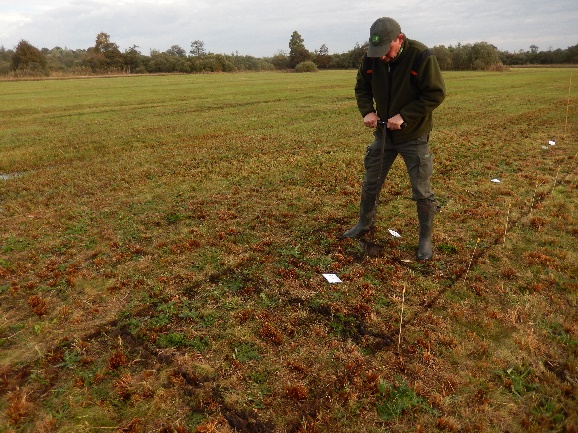

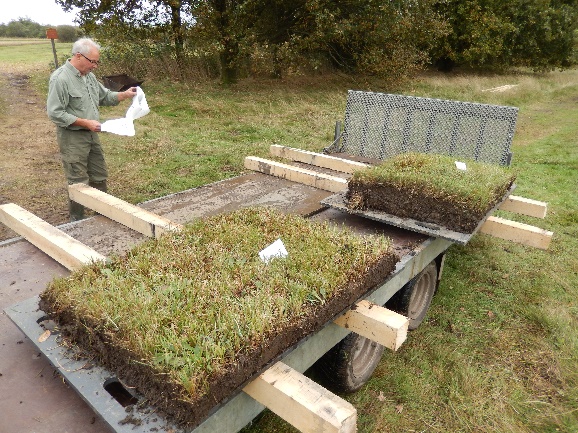


S1.D: The sod on the road plates is sorted according to their target location.


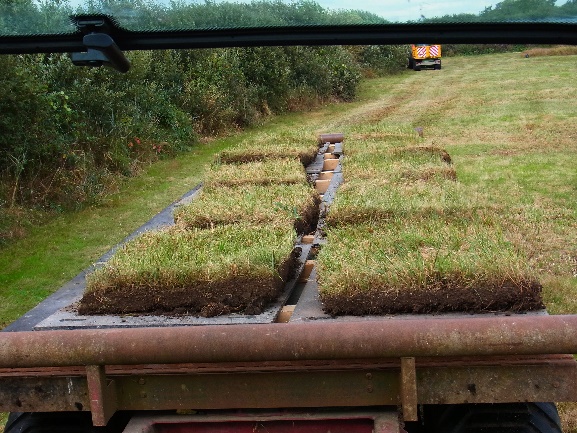

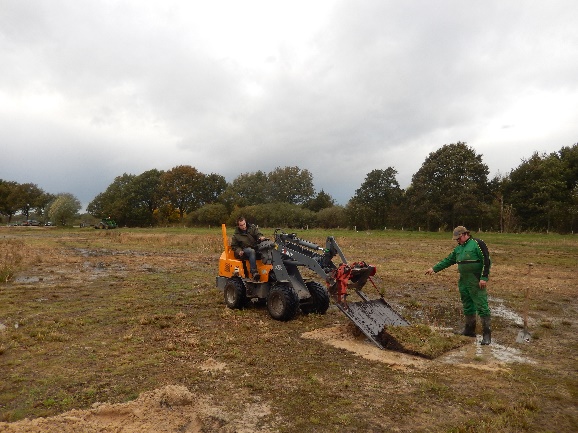

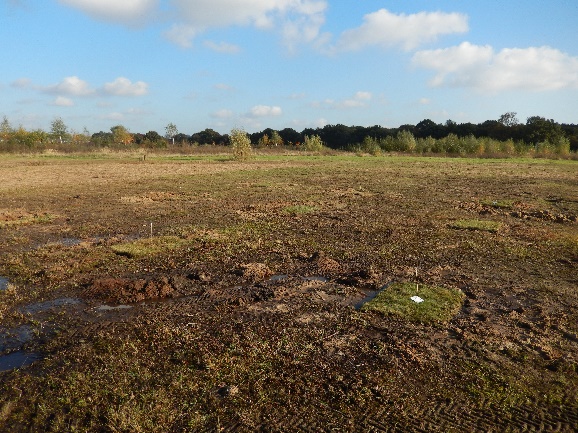


S1.G: A patch with nine sods after translocation to target location CG.

S1.E: The sods are transported to their target location.

S1.F: The sod glides into an earlier dug out ditch.

S1.H: Link to video of sod translocations in 2013: https://youtu.be/qU38yMohuP8

# **Supporting Information S2.** Average values of environmental factors and standard deviation (mean ± sd) in the three different treatments and four years of research. Covers are given in percentage, heights in cm. Nitrogen, moisture and pH are Ellenberg indicator values. Significant differences between years were tested using Dunn-Bonferroni post hoc test. Significant differences are denoted by different letters between years.

| *Sods* | **2014** | **2015** | **2016** | **2017** |
| --- | --- | --- | --- | --- |
| *Total Vegetation Cover* | 96.66 ± 4.8 ^a^ | 93.79 ± 6.6 ^b^ | 98.44 ± 2.5 ^a^ | 98.24 ± 4.6 ^a^ |
| *Shrub Cover* | 0.07 ± 0.2 ^a^ | 0.77 ± 2.4 ^ab^ | 1.04 ± 2.3 ^ab^ | 2.6 ± 5.5 ^b^ |
| *Herb Cover* | 82.27 ± 14.6 ^a^ | 92.96 ± 7.1^b^ | 98.11 ± 2.6 ^c^ | 97.5 ± 5.3 ^c^ |
| *Moss Cover* | 15.61 ± 17.6 | 9.4 ± 14.3 | 10.74 ± 14.2 | 10.37 ± 12.8 |
| *DOM Cover* | 8.46 ± 9.3 ^a^ | 27.59 ± 14.8 ^b^ | 7.18 ± 3.9 ^a^ | 23.42 ± 18.2 ^b^ |
| *Bare Soil Cover* | 4.70 ± 4.8 ^a^ | 2.07 ± 4.3 ^b^ | 1.81 ± 2.8 ^b^ | 0.92 ± 2.2 ^b^ |
| *Sanguisorba Cover* | - | 16.75 ± 16.6 | 18.31 ± 22.7 | 13.89 ± 15.3 |
| *Sanguisorba Number* | - | 27.72 ± 37.8 | 35.4 ± 41.1 | 7.81 ± 9.3 |
| *Mean Height* | 14.13 ± 4.5 ^a^ | 28 ± 9.4 ^b^ | 36.96 ± 13.8 ^c^ | 37.33 ± 14 ^c^ |
| *STDEV Height* | 5.08 ± 3.0 ^a^ | 9.45 ± 5.2 ^b^ | 14.46 ± 7.8 ^c^ | 16.2 ± 8 ^c^ |
| *Nitrogen* | 4.073 ± 0.5 | 4.07 ± 0.5 | 3.95 ± 0.3 | 3.95 ± 0.3 |
| *Moisture* | 7.03 ± 0.6 | 7.03 ± 0.6 | 7.16 ± 0.4 | 7.16 ± 0.3 |
| *pH* | 5.35 ± 0.6 | 5.35 ± 0.6 | 5.4 ± 0.5 | 5.4 ± 0.5 |
| *C controls* |  |  |  |  |
| *Total Vegetation Cover* | 53.75 ± 29.8 | 49.64 ± 26.2 | 59.73 ± 27.5 | 56.41 ± 29.8 |
| *Shrub Cover* | 3.27 ± 4.4 ^a^ | 12.56 ± 13.6 ^b^ | 7.21 ± 7.6 ^ab^ | 12.96 ± 14.8 ^b^ |
| *Herb Cover* | 25.85 ± 21.6 ^a^ | 35.42 ± 27.8 ^ab^ | 45.35 ± 25.8 ^b^ | 40.11 ± 29.53 ^b^ |
| *Moss Cover* | 30.12 ± 23.8 ^a^ | 14.25 ± 15.6 ^b^ | 21.73 ± 22.4 ^ab^ | 23.37 ± 23.31 ^ab^ |
| *DOM Cover* | 5.21 ± 6.4 ^a^ | 13.64 ± 8.3 ^b^ | 7.46 ± 7.3 ^a^ | 18.02 ± 13.0 ^b^ |
| *BareSoil Cover* | 46.79 ± 29.2 | 45.42 ± 27.9 | 42.00 ± 28.4 | 46.63 ± 31.50 |
| *Sanguisorba Cover* | - | 0.31 ± 1.2 | 0.17 ± 0.5 | 0.33 ± 1.2 |
| *Sanguisorba Number* | - | 0.10 ± 0.47 | 0.46 ± 1.7 | 0.17 ± 0.9 |
| *Mean Height* | 4.43 ± 4.2 ^a^ | 11.87 ± 8.6 ^b^ | 16.22 ± 16.1 ^b^ | 17.26 ± 15.14 ^b^ |
| *STDEV Height* | 3.80 ± 3.6 ^a^ | 9.98 ± 6.9 ^b^ | 10.25 ± 7.1 ^b^ | 10.55 ± 5.2 ^b^ |
| *Nitrogen* | 4.20 ± 0.5 ^a^ | 4.20 ± 0.5 ^a^ | 3.88 ± 0.4 ^b^ | 3.89 ± 0.4 ^b^ |
| *Moisture* | 6.77 ± 0.5 | 6.77 ± 0.5 | 6.92 ± 0.4 | 6.92 ± 0.4 |
| *pH* | 5.10 ± 0.4 ^a^ | 5.10 ± 0.4 ^a^ | 5.29 ± 0.4 ^b^ | 5.27 ± 0.4 ^b^ |
| *O controls* |  |  |  |  |
| *Total Vegetation Cover* | - | 69.58 ± 23.3 ^a^ | 84.06 ± 18.5 ^b^ | 75.89 ± 21.2 ^b^ |
| *Shrub Cover* | - | 9.87 ± 12.6 | 7.92 ± 9 | 14.1 ± 15.1 |
| *Herb Cover* | - | 51.15 ± 26.2 | 57.92 ± 17.8 | 59.68 ± 25 |
| *Moss Cover* | - | 34.71 ± 26.1 ^a^ | 59.46 ± 31 ^b^ | 48.75 ± 29.6 ^b^ |
| *DOM Cover* | - | 26.56 ± 18.2 ^a^ | 8.08 ± 7 ^b^ | 15.14 ± 9.7 ^c^ |
| *BareSoil Cover* | - | 18.75 ± 21 | 15.27 ± 18.4 | 22.91 ± 22.3 |
| *Sanguisorba Cover* | - | 1.35 ± 3 | 1.02 ± 1.6 | 1.89 ± 3.0 |
| *Sanguisorba Number* | - | 0.6 ± 1.6 | 1.37 ± 2.4 | 1.12 ± 1.9 |
| *Mean Height* | - | 14.02 ± 8.6 ^a^ | 18.29 ± 10.1 ^b^ | 17.25 ± 9.3 ^ab^ |
| *STDEV Height* | - | 8.86 ± 5.5 ^a^ | 11.47 ± 5.6 ^b^ | 10.38 ± 5.6 ^ab^ |
| *Nitrogen* | - | - | 3.86 ± 0.3 | 3.86 ± 0.3 |
| *Moisture* | - | - | 6.86 ± 0.6 | 6.85 ± 0.6 |
| *pH* | - | - | 5.26 ± 0.4 | 5.26 ± 0.4 |

**Supporting Information S3.** Graphs of average values with standard errors in the three different treatments (sods, c-controls and o-controls) in 2017. Graph A corresponds to total coverage of vegetation (%), graph B corresponds to coverage bare soil (%), graph C corresponds to vegetation height (cm) and graph D corresponds to the coverage of Sanguisorba officinalis.
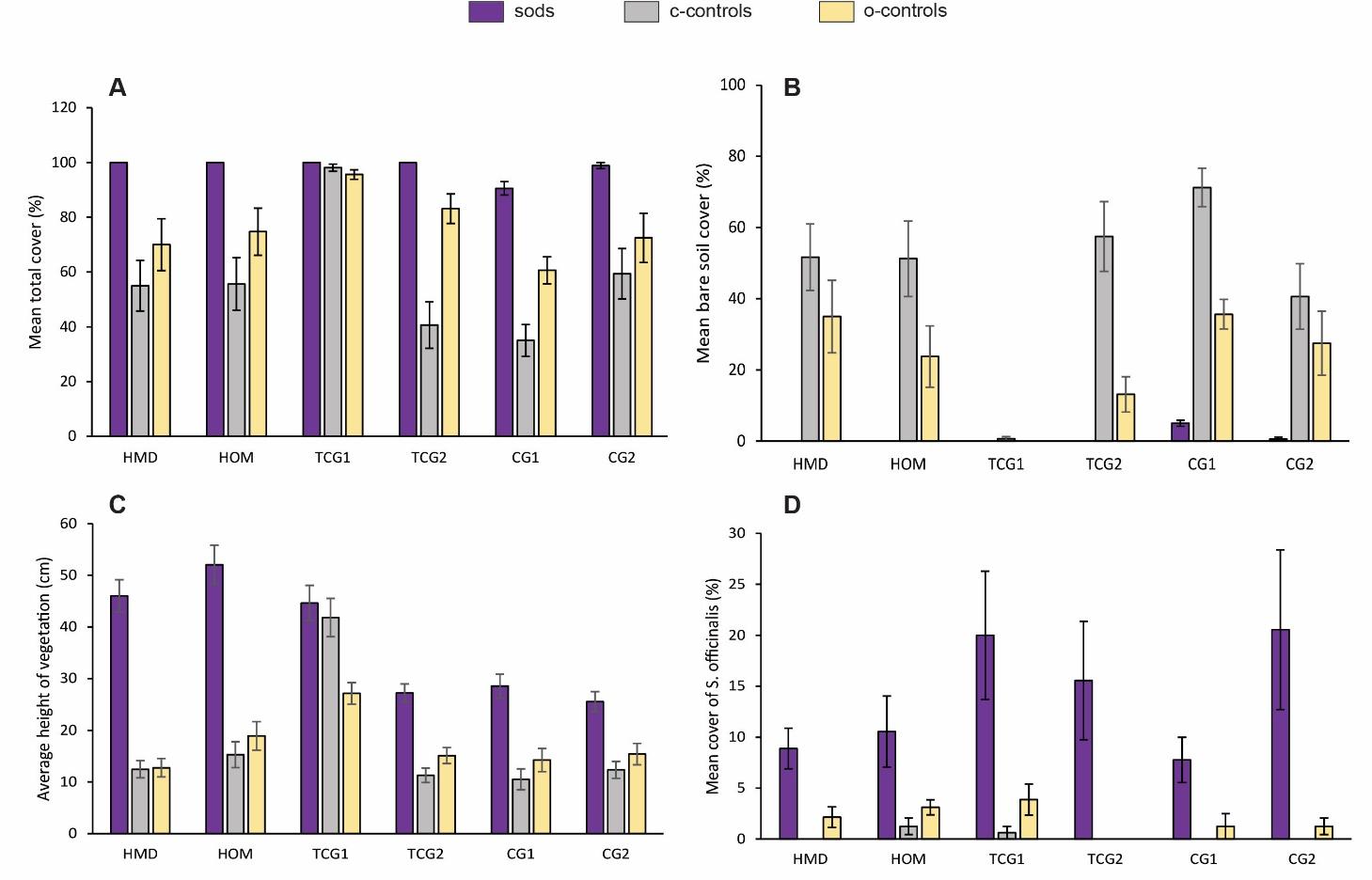


# **Supporting Information S4.** List of ant species by treatment and year. The numbers of sods or control plots occupied by the ant species are given. C-controls and o-controls have a distance of 3 m and >10 m to the plots respectively.

| Ant species | 2014 | |  | 2015 | | |
| --- | --- | --- | --- | --- | --- | --- |
|  | sods | c-control |  | sods | c-control | o-control |
| *Myrmica scabrinodis* | 19 | 0 |  | 19 | 15 | 1 |
| *Myrmica rubra* | 0 | 0 |  | 0 | 1 | 0 |
| *Myrmica ruginodis* | 0 | 0 |  | 0 | 2 | 0 |
| *Myrmica gallienii* | 4 | 3 |  | 3 | 0 | 0 |
| *Myrmica sabuleti* | 2 | 0 |  | 2 | 0 | 0 |
| *Myrmica rugulosa* | 1 | 0 |  | 0 | 0 | 0 |
| *Lasius niger* | 11 | 11 |  | 15 | 11 | 11 |
| *Lasius umbratus* | 1 | 0 |  | 0 | 0 | 0 |
| All *Myrmica* species | 24 | 3 |  | 22 | 17 | 1 |
| All ant species | 28 | 11 |  | 36 | 25 | 12 |

| Ant species | 2016 | | |  | 2017 | | |
| --- | --- | --- | --- | --- | --- | --- | --- |
|  | sods | c-control | o-controls |  | sods | c-control | o-control |
| *Myrmica scabrinodis* | 7 | 7 | 7 |  | 18 | 6 | 12 |
| *Myrmica rubra* | 0 | 0 | 2 |  | 0 | 0 | 1 |
| *Myrmica ruginodis* | 0 | 0 | 0 |  | 0 | 0 | 0 |
| *Myrmica gallienii* | 5 | 2 | 0 |  | 6 | 3 | 1 |
| *Myrmica sabuleti* | 1 | 0 | 0 |  | 1 | 1 | 0 |
| *Myrmica rugulosa* | 0 | 1 | 2 |  | 0 | 0 | 0 |
| *Myrmica schencki* | 1 | 0 | 0 |  | 0 | 0 | 0 |
| *Lasius niger* | 14 | 12 | 14 |  | 17 | 11 | 11 |
| *Lasius umbratus* | 0 | 0 | 0 |  | 0 | 0 | 0 |
| *Lasius flavus* | 0 | 1 | 0 |  | 0 | 0 | 0 |
| All *Myrmica* species | 13 | 10 | 9 |  | 25 | 10 | 11 |
| All ant species | 26 | 20 | 20 |  | 41 | 20 | 22 |

#
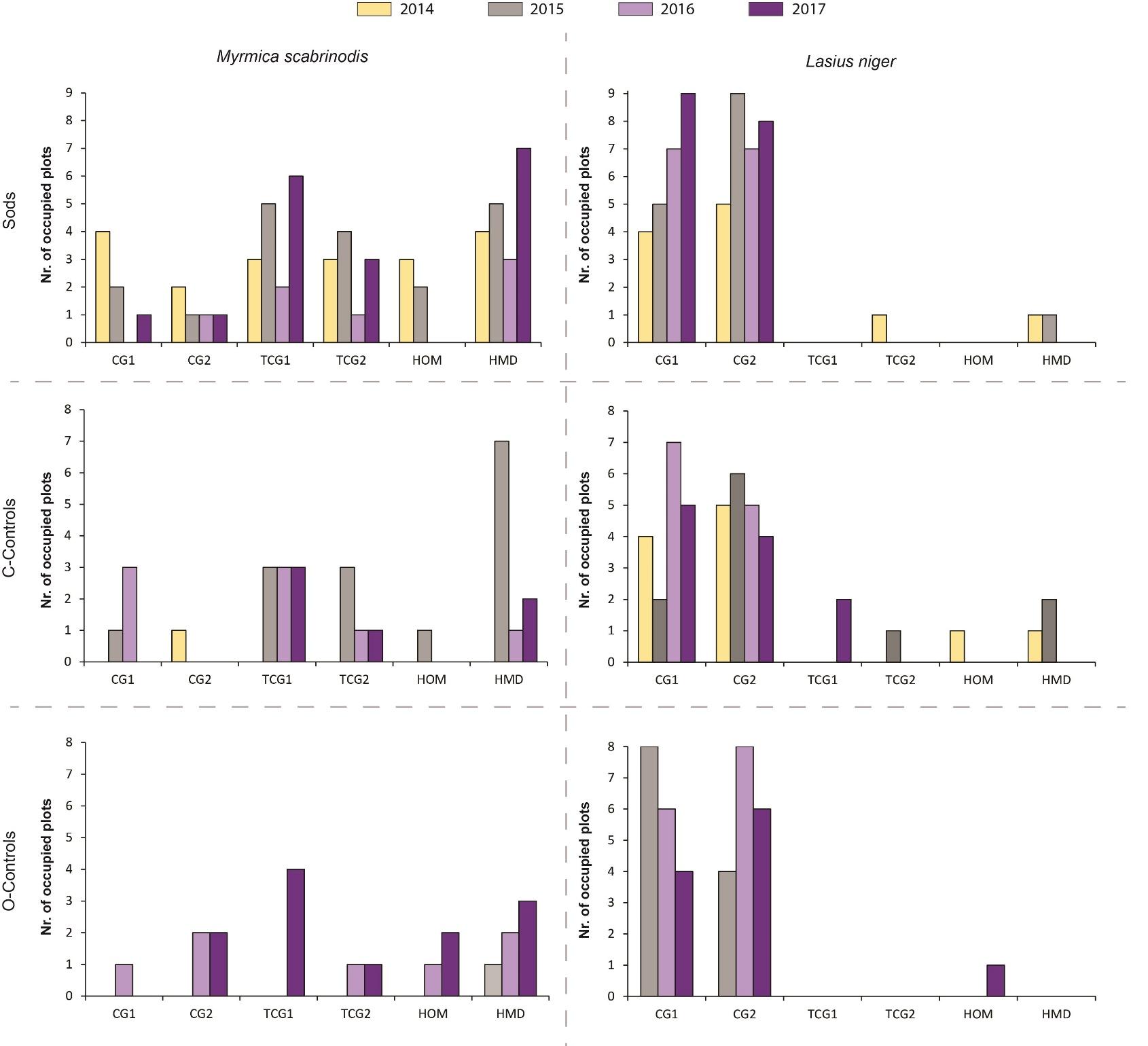
**Supporting Information S5a.** Number of plots occupied by *Myrmica scabrinodis* (left-hand column) and *Lasius niger* (right-hand column) in sods (upper row), c-controls (middle) and o-controls (bottom) in four years in six sites. Total number of plots in each treatment: 9 of sods, 8 of c-controls and 8 of o-controls.

#
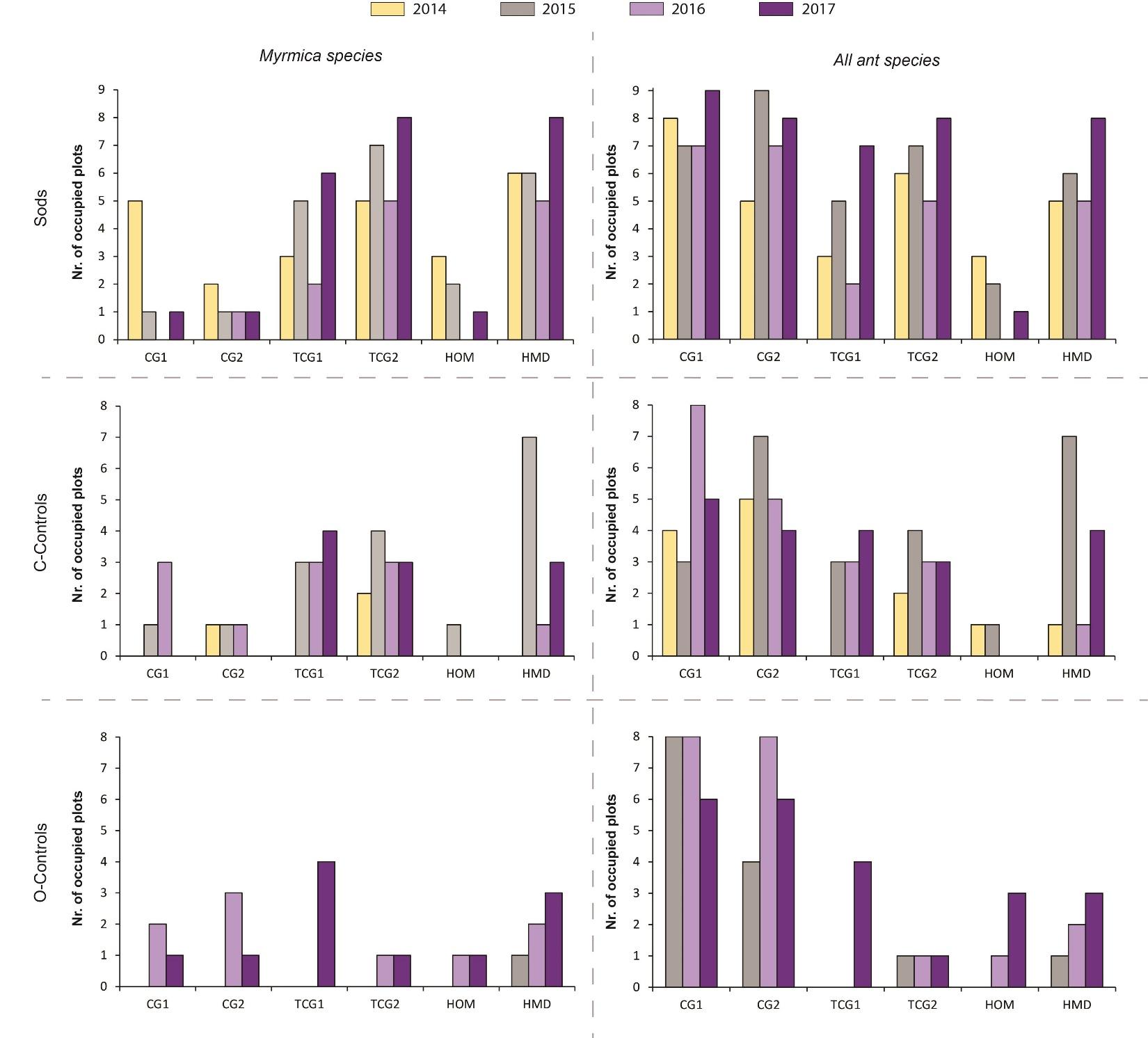
**Supporting Information S5b.** Number of plots occupied by *Myrmica* species (left-hand column) and all ant species (right-hand column) in sods (upper row), c-controls (middle) and o-controls (bottom) in four years in six sites. Total number of plots in each treatment: 9 of sods, 8 of c-controls and 8 of o-controls.

# **Supporting Information S6.** Graph of Pearson correlations between environmental factors. Higher values are shown in stronger colour tones (red and blue). N= 572.
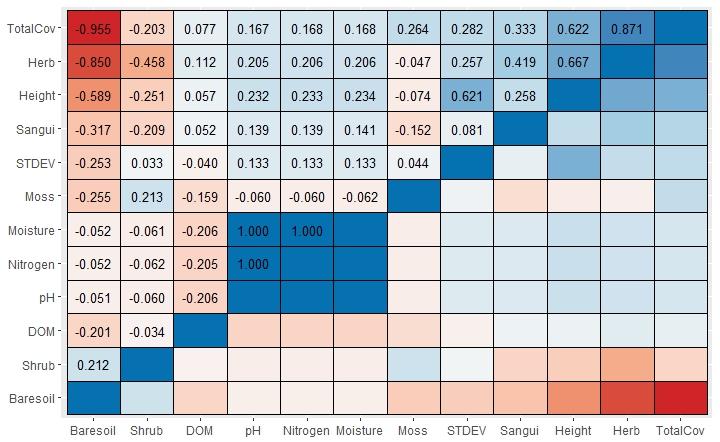


# **Supporting Information S7.** Generalized linear mixed model results of all *Myrmica* species’ and all ant species’ presence based on various influential factors after the sod translocation in 2013. Each row refers to one independent variable (one model per row) for the all Myrmica species’ presence. In the columns appear AICs, Estimate, StE (standard error), z (z value) and adjusted p-value. All variables have 1 degree of freedom; † is a categorical variable. Adjusted P-values according to Benjamini-Hochberg procedure, * for significant values (FDR=0.1). Cover variables have N=550 except Ellenberg indicator values, N=502 and Sanguisorba Cover, N=448.

|  |  | All *Myrmica* species | | | | |  | All ant species | | | | |
| --- | --- | --- | --- | --- | --- | --- | --- | --- | --- | --- | --- | --- |
|  | Model | AIC | Estimate | St E | z | Adjusted P-value | Model | AIC | Estimate | St E | z | Adjusted P-value |
| *N=550* | Herb Cover | 582.0 | 0.71 | 0.12 | 5.88 | 0,007* | Year of excavation^†^ | 671.5 | - | 0.19 | - | 0,007* |
|  | Total Veg. Cover | 588.0 | 0.70 | 0.14 | 5.18 | 0,014* | Bare soil Cover | 748.7 | -0.35 | 0.09 | -3.88 | 0,023* |
|  | Bare soil Cover | 588,0 | -0.72 | 0.14 | -5.11 | 0,021* | Total Veg. Cover | 750.7 | 0.33 | 0.09 | 3.64 | 0,031* |
|  | Year of excavation^†^ | 596.7 | - | 0.23 | - | 0,029* | Herb Cover | 754.7 | 0.27 | 0.08 | 3.12 | 0,038* |
|  | *Lasius niger* | 610.9 | -0.88 | 0.27 | -3.25 | 0,036* | Shrub Cover | 758.0 | 0.23 | 0.09 | 2.53 | 0,053 |
|  | Shrub Cover | 614.7 | -0.33 | 0.13 | -2.64 | 0,050* | Moss Cover | 760.2 | -0.18 | 0.08 | -2.08 | 0,061* |
|  | Mean Height | 617.8 | 0.22 | 0.10 | 2.28 | 0,064 | DOM Cover | 761.6 | 0.16 | 0.09 | 1.72 | 0,069* |
|  | DOM Cover | 621.0 | 0.14 | 0.10 | 1.43 | 0,071 | Mean Height | 763.8 | 0.08 | 0.09 | 0.91 | 0,084 |
|  | Stdev Height | 621.4 | -0.13 | 0.11 | -1.23 | 0,079 | Stdev Height | 764.5 | -0.02 | 0.09 | -0.25 | 0,100 |
|  | Moss Cover | 622.8 | -0.04 | 0.10 | -0.39 | 0,100 | *Lasius niger* | - | - | - | - | - |
| *N=502* | Ellenberg Moisture | 585.8 | 0.27 | 0.11 | 2.50 | 0,057 | Ellenberg Nitrogen | 680.9 | -0.42 | 0.10 | -4.29 | 0,015* |
|  | Ellenberg pH | 591.2 | -0.11 | 0.10 | -1.08 | 0,086 | Ellenberg pH | 699.7 | 0.09 | 0.09 | 0.99 | 0,07 |
|  | Ellenberg Nitrogen | 592.2 | -0.04 | 0.10 | -0.41 | 0,093 | Ellenberg Moisture | 700.5 | -0.04 | 0.09 | -0.42 | 0,092 |
| *N=448* | *Sanguisorba* Cover | 495.5 | 0.31 | 0.10 | 3.04 | 0,043* | *Sanguisorba* Cover | 616.2 | 0.29 | 0.10 | 2.80 | 0,046* |
